# Home Parenteral Support in Severe Gitelman Syndrome: A Case Report

**DOI:** 10.1002/ccr3.73210

**Published:** 2026-07-22

**Authors:** Georgia Martin, Ibrahim Fahal, Shameer Mehta

**Affiliations:** ^1^ Queen Mary University of London London UK; ^2^ Department of Nephrology Barking Havering and Redbridge University Hospitals NHS Trust Romford UK; ^3^ Department of Gastroenterology Barts Health NHS Trust London UK

**Keywords:** catheter‐related bloodstream infection, Gitelman syndrome, home parenteral support, hypokalaemia, hypomagnesemia

## Abstract

Gitelman syndrome (GS), also known as familial hypokalaemia‐hypomagnesemia, is a rare autosomal recessive renal tubular disorder characterized by hypokalaemia, hypomagnesemia, metabolic alkalosis, and hypocalciuria. Electrolyte replacement of potassium and magnesium remains the mainstay of management, with oral supplementation achieving this in the majority of patients. We report the case of a 65‐year‐old woman with a severe manifestation of GS, managed with high‐dose intravenous electrolyte replacement as part of a home parenteral support (HPS) regimen in addition to oral supplementation. Initiation of HPS, whilst life‐saving and life‐prolonging, also negatively impacted her quality of life and was complicated by multiple catheter‐related bloodstream infections (CRBSI) and catheter‐related venous thromboses (CRVT). After 15 years of HPS, her management was transitioned to a shared care model under an accredited multidisciplinary intestinal failure (IF) unit. Her significant parenteral electrolyte requirement was subsequently rationalized, and provision of compounded home infusions reduced her treatment burden by allowing her to sleep through each night without additional connections and disconnections. No further episodes of CRBSIs occurred, and no further venous catheter exchanges were necessary. In this case report, we illustrate the presentation of severe GS, highlight the necessity of daily intravenous electrolyte replacement in such cases, and emphasize how collaborative management between nephrology and teams experienced in long‐term catheter care can be crucial in optimizing treatment, improving patient outcomes, and enhancing quality of life.

## Key Clinical Message

Long‐term central venous catheters carry an infection risk and may contribute to venous thrombosis development, potentially compromising future venous access and predisposing to thromboembolisms. In conditions where prolonged intravenous therapy is not typically used, collaborative involvement of specialist units experienced in long‐term catheter care can significantly improve patient outcomes.

## 1 Introduction

Gitelman syndrome (GS) is a rare salt‐wasting tubulopathy, with a reported incidence of ~1 in 40,000 people [1]. The condition usually presents during adolescence, with the average age of onset of 20 years [2]. A recent study reported an earlier onset of symptoms in men, with an increased prevalence of the disease in males in childhood and females in adulthood [3]. The symptom burden was also shown to be higher in females. The incidence of GS is higher in the East Asian population [4]. GS is most commonly the result of a biallelic loss‐of‐function mutation in the SLC12A3 gene, which encodes the sodium‐chloride cotransporter within the renal distal convoluted tubule [5]. The majority of these mutations are missense, accounting for ~59% of genetic variants [6]. In a small subset of patients, mutations have been found in the chloride channel CLCNKB gene [7]. Due to these mutations, renal salt loss occurs, resulting in hypokalaemia, hypomagnesemia, and hypocalciuria, alongside metabolic alkalosis [8]. Increased activation of the renin‐angiotensin system can also be seen in some patients.

The clinical presentation of GS is highly heterogeneous. Some individuals remain completely asymptomatic and are diagnosed incidentally, while others experience mild symptoms related to fluid and electrolyte imbalance [9]. These symptoms can include salt craving, polydipsia, polyuria, muscle weakness, and fatigue. An important differential diagnosis to consider is Bartter syndrome, another salt‐wasting tubulopathy, where a gene mutation affects salt reabsorption in the thick ascending limb of Henle [9]. Diagnosis of Bartter syndrome is often earlier than GS, and growth retardation is commonly present, which helps to distinguish between the two salt‐wasting tubulopathies [9]. Additionally, urinary calcium levels are typically normal or high in Bartter syndrome, whereas in GS, they are low. Genetic testing provides a definitive diagnosis between the two, with a variant in the SLC12A3 gene confirming GS. Replacement of electrolyte losses via oral supplementation is the cornerstone of GS management, in addition to a diet high in salt [10]. Pharmacological therapy includes potassium‐sparing diuretics and angiotensin system blockers. Despite the availability of these treatment options, in a proportion of patients, severe and potentially life‐threatening complications due to electrolyte disturbances can occur. Hypokalaemia and hypomagnesaemia can result in potentially fatal cardiac arrhythmias such as polymorphic ventricular tachycardia and ventricular fibrillation, both of which have been reported [11, 12]. Long‐term sequelae of GS can include chondrocalcinosis, present in 20%–50% of patients, and chronic kidney disease [10].

## 2 Case History

The care of our 65‐year‐old patient was initially overseen by a nephrology team after she presented at their hospital at the age of 46 with symptoms of weakness and dizziness. She had been diagnosed with GS 2 years earlier at a nearby hospital and had a background of type 2 diabetes mellitus, diagnosed 8 years prior, which she managed with insulin. Initial treatment of her GS consisted of oral potassium and magnesium. For the 2 years following her initial presentation, she required A&E attendance every 6–8 weeks due to persistent, refractory electrolyte disturbances. She was subsequently commenced on home parenteral support (HPS) by her nephrology team. She received intravenous potassium and magnesium, in addition to ongoing oral supplementation, to maintain her electrolyte balance. Her HPS regimen consisted of four separate daily infusions, including nightly connections and disconnections at midnight, which affected her ability to sleep through the night. The infusions were used exclusively for electrolyte supplementation as she maintained a normal oral intake. She remained on HPS for 15 years, during which time she required 22 venous catheters. Her long‐term intravenous therapy was complicated by 18 catheter‐related bloodstream infections (CRBSI), all requiring line replacement, resulting in 15 episodes of catheter‐related venous thromboses (CRVT). These events led to repeated sepsis‐associated acute kidney injury, cumulative nephron loss, and progressive chronic kidney disease.

Following NHS England's intestinal failure (IF) service reorganization, the regional accredited IF service assumed joint responsibility for her care in 2023. At this time, she was receiving a total of 2.2 L of intravenous fluid, via four separate infusions over a period of 14 h, containing a total of 80 mmol of potassium and 40 mmol of magnesium. This was administered via a single‐lumen tunneled Hickman line, which had been in situ for 8 months. In addition, she was taking seven Sando‐K tablets four times daily, providing approximately 324 mmol of oral potassium per day. Her medications also included two potassium‐sparing diuretics, spironolactone 25 mg once daily and amiloride 5 mg once daily. At this time, she was not receiving any oral magnesium supplementation.

## 3 Therapeutic Intervention

At her initial review by the IF team, in collaboration with her nephrology team, her HPS regimen was rationalized by consolidating all fluids and electrolytes into a single bespoke infusion, administered overnight over 12 h, without altering the total daily electrolyte content. This approach aimed to reduce catheter manipulations, thereby minimizing infection risk and reducing sleep disruption. Prophylactic daily antimicrobial catheter lock therapy with taurolidine, a solution with both antimicrobial and anticoagulant properties, was subsequently introduced.

Over the 6 months following her referral, her oral potassium supplementation was gradually reduced due to significant gastrointestinal discomfort, with a corresponding increase in intravenous potassium replacement to maintain adequate serum levels. By the end of this period, her parenteral potassium requirement had increased to 160 mmol per infusion, while her oral intake had been reduced to four Sando‐K tablets three times daily.

During this period of time, the patient also reported worsening gastro‐esophageal reflux symptoms and, as such, her omeprazole dose was increased from 20 to 40 mg once daily. Shortly thereafter, she developed muscle cramps and spasms. These symptoms were presumed to be secondary to hypomagnesaemia in the context of increased proton pump inhibitor therapy, although this was not definitively proven. Contingency infusions of magnesium were prescribed. Due to the muscle spasms and cramps persisting, delivery of parenteral magnesium was increased to 48 mmol daily with three additional 10 mmol infusions per week. Oral magnesium supplementation was then subsequently introduced, although this was poorly tolerated due to diarrhea and abdominal discomfort. Increasing the magnesium content within her parenteral support was discussed. However, doing so would have required an increase in the overall intravenous fluid volume and, as such, she declined. She therefore continued oral magnesium supplementation despite some gastrointestinal side effects.

**FIGURE 1:**
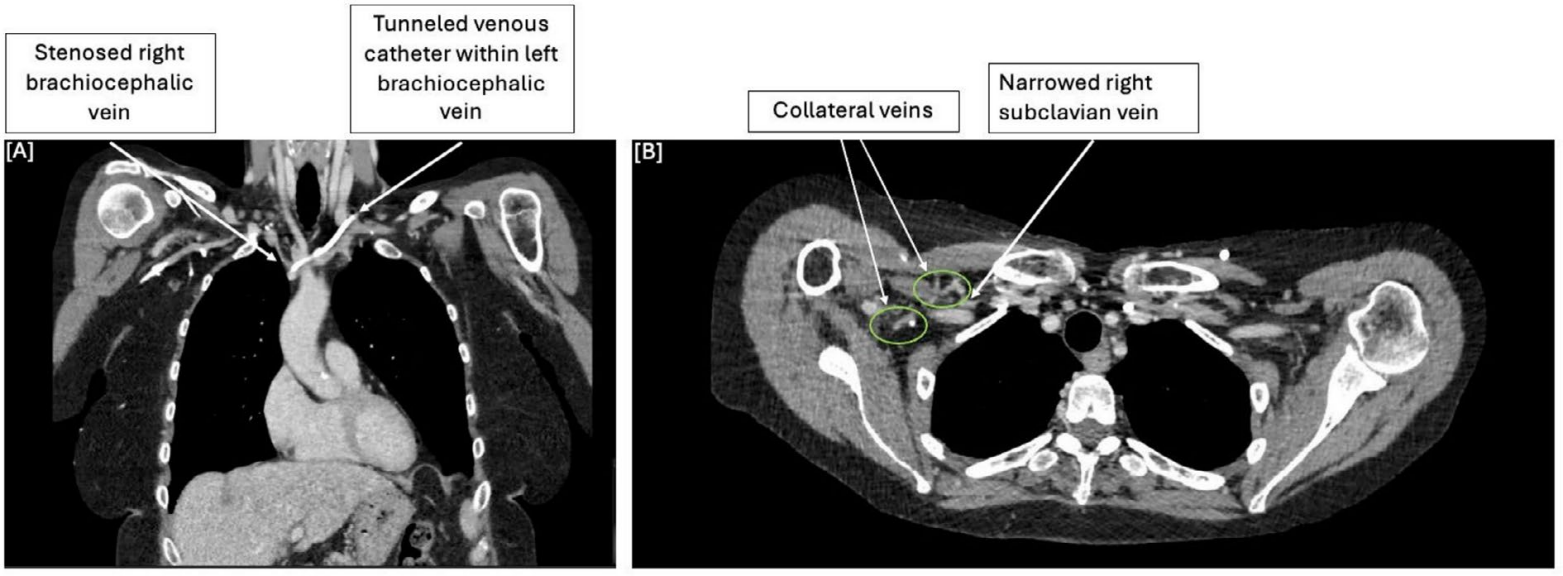
CT venography demonstrating central venous stenosis with collateralisation. (A) Coronal plane showing the current position of the catheter within the left brachiocephalic vein and stenosis of the right brachiocephalic vein. (B) Transverse plane showing stenosis of the right subclavian vein with formation of collateral veins.

Nine months post‐referral, the patient described symptoms of venous occlusion, specifically positional headaches that were occurring upon lying flat. She also noticed distention of the veins over her chest wall and left side of her neck, which worsened during parenteral infusions. The patient did not report any neck, facial, or upper chest oedema. On clinical examination, visible collateral veins were present on the anterior chest wall. Computed tomography (CT) venography of the neck and chest demonstrated chronic occlusion of the right internal jugular, right subclavian, and right brachiocephalic veins, with extensive collateralisation involving the right chest wall and neck. Figure 1 below shows the CT venography images, demonstrating the current position of the catheter within the left brachiocephalic vein, stenosis of the right subclavian and brachiocephalic veins, and the formation of collateral veins.

In the context of the patient's declining renal function due to her chronic kidney disease, it was discussed that renal replacement therapy might be required in the future. Due to her existing extensive CRVTs, central venous angioplasty was planned to optimize venous outflow in case an arteriovenous fistula would be required in the future.

## 4 Outcomes

Through close joint working between the nephrology and multidisciplinary IF teams, several modifications were made to this patient's management. A single bespoke parenteral infusion was formulated, and the intravenous component of her potassium supplementation was increased, allowing a reduction in her oral supplementation. This had a significant positive impact on her quality of life, as she was no longer required to wake during the night to change her infusions, and gastrointestinal side effects of oral potassium were reduced. The implementation of a taurolidine lock for catheter prophylaxis alongside the reduced number of daily infusions resulted in a meaningfully lower catheter infection risk. Notably, as a result, no further CRBSIs occurred, and no catheter replacements were required during the 18 months following referral. Previous episodes of CRVT, however, resulted in critical venous occlusions in major neck vessels, complicating the management of her coexisting chronic kidney disease and thus resulting in the need for a central venous angioplasty.

## 5 Discussion

To our knowledge, we describe the only instance of a patient in the UK with severe GS that requires HPS. A diet high in salt alongside potassium and magnesium supplementation remains the cornerstone of GS management, with a recent cohort study reporting that 94% of patients required potassium supplementation and 50% required magnesium supplementation [3]. In most cases, supplementation is administered orally, with intravenous replacement reserved for patients who are unable to tolerate oral therapy or who present with acute, severe complications of hypokalaemia or hypomagnesaemia, often triggered by gastrointestinal illness [10]. Longer‐term intravenous electrolyte therapy in GS is primarily reported during pregnancy, which is known to increase requirements, with a systematic review reporting that 37.9% of patients required IV supplementation at some point during gestation [13]. However, unique to this case, our patient had required long‐term intravenous potassium and magnesium in the form of HPS to manage her condition, in addition to oral potassium and two potassium‐sparing diuretics. This not only highlights the severity of her disease but also the challenges associated with managing refractory electrolyte disturbances in severe GS. Practical considerations for IF teams involved in the management of patients with severe GS are summarized in Box 1.

Long‐term intravenous replacement therapy necessitates highly specialized management to ensure formulations that successfully maintain electrolyte levels while minimizing the impact of treatment on daily life. Prior to referral, this patient's HPS regimen was daily, involving four sequential infusions with three line changes—including one at midnight—severely disrupting sleep. Due to specialist commissioning and contracting, accredited IF services are able to deliver bespoke HPS solutions that integrate all necessary electrolytes and minimize line handling and, as a result, both improve clinical stability and reduce treatment burden. This relies on close multidisciplinary working between specialist pharmacists, dietitians, and clinical nurse specialists.

The complexity in HPS management directly impacts infection risk and subsequently vascular access. Our patient experienced 18 CRBSIs over 14 years, corresponding to a rate of approximately 3.52 CRBSIs per 1000 catheter‐days. This is notably higher than the 0.31 per 1000 catheter‐days reported in specialist IF units, reflecting the additional expertise that such units contribute specifically in the area of catheter infection prevention [14]. While some CRBSIs can be treated with antibiotics and catheter salvage, this was not achieved in this patient, and each of the 18 episodes required line removal rather than conservative management. Salvage is generally not advised or is likely to fail when infections are caused by virulent or biofilm‐forming organisms (e.g., *Staphylococcus aureus* , *Pseudomonas* species, or fungal isolates such as *Candida* species), when there is exit‐site or tunnel‐tract infection, or when the patient is clinically unstable with sepsis. Such circumstances account for the majority of non‐salvaged catheters, even within specialist IF units. In this patient, the recurrent nature of the infections in a single long‐term tunneled catheter, together with the organisms and access‐site involvement encountered, precluded catheter salvage on each occasion. Several factors may have played a role in the development of these infections. The absence of a bespoke infusion bag necessitated multiple line connections and disconnections, thereby increasing the infection risk. In addition, the subsequent introduction of a taurolidine lock provided important additional protection. Taurolidine locks, which contain the amino acid taurolidine in addition to citrate, offer antimicrobial activity and biofilm eradication [15]. A randomized controlled trial demonstrated that using 1.35% taurolidine significantly reduces the rate of recurrent CRBSIs and decreases central venous catheter removals due to CRBSIs, compared with 0.9% saline, in adults with chronic IF dependent on HPS [16]. Following involvement of the IF team, she continued with a tunneled line, received a bespoke bag to reduce line handling, and was managed with a taurolidine lock, and consequently remained infection‐free for 18 months.

This patient also had 15 CRVTs and, although the risk factors for CRVT are not fully elucidated, those reported in the literature include infection and the number of insertion attempts, both of which are relevant in this case [17, 18]. The resultant CRVTs eventually led to critical venous occlusion, leading to the requirement for central venoplasty in order to preserve future access. Thromboses such as CRVTs are a serious complication in patients receiving HPS, as they can compromise central venous access, as well as predispose the patient to thromboembolic events. For patients with chronic IF who rely on HPS in the form of parenteral nutrition, CRVTs involving two or more central veins may prompt consideration of intestinal transplantation [19]. This consideration applies specifically to patients with irreversible IF who have exhausted their central venous access. It does not apply to GS, in which the bowel is structurally and functionally normal. Intestinal transplantation would neither be indicated nor address the renal tubular basis of the electrolyte losses in GS, and should not be regarded as a therapeutic option in this setting. Renal transplantation in GS has, by contrast, been reported only at the level of a single case report, and its role in this condition therefore remains anecdotal [20]. Consequently, in patients with GS who are dependent on HPS, preservation of long‐term central venous access is an overriding priority, as loss of access may have serious and potentially life‐threatening consequences. Where such access becomes jeopardized, formation of an arteriovenous fistula warrants consideration as a durable route for fluid therapy with potassium and magnesium supplementation, analogous to its established role in hemodialysis. Management should accordingly be directed toward strict central line care protocols and access‐preserving strategies rather than escalation to transplantation.

Although this patient represents a rare case of GS due to her intravenous electrolyte requirements, there is a growing cohort of patients without IF who are reliant on HPS [21]. The challenges outlined in this case report highlight the importance of close working between nephrology and IF teams, including multidisciplinary support. As such, similar principles should therefore be applied to other patients who necessitate management with HPS, with coordinated care between primary specialty teams and IF services. Despite many healthcare professionals having experience in long‐term catheter care, the clinical requirements, risk profiles, and potential complications are particularly distinct in patients receiving HPS. Specialist units offer bespoke HPS formulations with a particular focus on infection prevention. Early involvement of such units is therefore essential to improve quality of life, reduce mortality risk from CRBSIs, protect future venous access, and, ultimately, provide the individualized treatment plans required by this group of patients.

BOX 1 **Practical Considerations for Intestinal Failure Teams.**

Patients requiring HPS for reasons other than IF are increasingly being managed by IF teams, and the underlying cause of refractory electrolyte loss may not initially be apparent. The following structured approach is offered to aid recognition of GS and to guide escalation of electrolyte supplementation in such patients.

*When to consider Gitelman syndrome*

1. Persistent hypokalaemia and hypomagnesaemia with metabolic alkalosis that is disproportionate to, or unexplained by, gastrointestinal losses or the degree of intestinal failure.
2. Inappropriately high urinary potassium and magnesium excretion despite low serum levels, together with low urinary calcium and normal or low blood pressure.
3. Electrolyte disturbances that are refractory to standard oral replacement, or that require recurrent hospital attendance for intravenous correction.
4. Absence of an alternative explanation (for example, diuretic use, vomiting, or a high‐output stoma), prompting referral for genetic testing. A biallelic SLC12A3 variant confirms the diagnosis and distinguishes GS from Bartter syndrome.

*Escalation of electrolyte supplementation*

1. Optimize oral potassium and magnesium replacement alongside a liberal salt intake as first‐line therapy.
2. Add potassium‐sparing diuretics (for example, spironolactone or amiloride) and, where appropriate, renin‐angiotensin system blockade to reduce renal potassium wasting.
3. Correct magnesium early and adequately, as hypomagnesaemia perpetuates refractory hypokalaemia. Review and minimize contributory drugs, in particular proton pump inhibitors.
4. Where oral therapy is not tolerated or fails to maintain safe serum levels, introduce intravenous replacement. If a chronic intravenous requirement is established, refer to an accredited IF unit for structured HPS.
5. Consolidate replacement into a single bespoke compounded infusion to minimize catheter handling, and institute antimicrobial catheter lock prophylaxis to reduce infection risk.

*Red flags in the acute admissions setting.*

When a patient with severe GS dependent on HPS presents acutely, the following features should prompt urgent assessment and early involvement of both the nephrology and IF teams:

1. Symptoms or signs of severe hypokalaemia or hypomagnesaemia (profound muscle weakness, cramps, tetany, palpitations, or syncope) which may herald life‐threatening cardiac arrhythmias. Obtain an urgent ECG and institute continuous cardiac monitoring.
2. Fever, rigors, or systemic upset in a patient with a central venous catheter suggestive of CRBSI. Take peripheral and catheter‐drawn blood cultures before antimicrobials and avoid unnecessary catheter removal until discussed with the specialist team.
3. Positional headache, facial or upper‐limb swelling, or distended chest‐wall veins suggesting central venous stenosis or thrombosis. Arrange appropriate venous imaging and protect any remaining access.
4. Reduced urine output or rising creatinine, indicating acute kidney injury superimposed on chronic kidney disease, which may acutely worsen electrolyte handling.
5. Interruption of the parenteral infusion for any reason, as even short breaks in supplementation can precipitate dangerous electrolyte depletion. Do not stop or alter the bespoke infusion without specialist advice.

## Author Contributions

**Ibrahim Fahal:** writing – review and editing. **Georgia Martin:** conceptualization, writing – original draft, writing – review and editing. **Shameer Mehta:** conceptualization, supervision, writing – review and editing.

## Funding

The authors have nothing to report.

## Consent

Written informed consent was obtained from the patient for publication of this case report.

## Conflicts of Interest

The authors declare no conflicts of interest.

## Data Availability

Data sharing not applicable to this article as no datasets were generated or analyzed during the current study.
